# Host Plant Associations of an Entomopathogenic Variety of the Fungus, *Colletotrichum acutatum*, Recovered from the Elongate Hemlock Scale, *Fiorinia externa*

**DOI:** 10.1673/031.009.2501

**Published:** 2009-05-29

**Authors:** José A. P. Marcelino, Svetlana Gouli, Bruce L. Parker, Margaret Skinner, Lora Schwarzberg, Rosanna Giordano

**Affiliations:** ^1^ Department of Plant and Soil Sciences, Entomology Research Laboratory, University of Vermont, Burlington, Vermont, 05405-0105 USA; ^2^ New York State Department of Environmental Conservation, Albany, New York, 12233-1750 USA; ^3^ Illinois Natural History Survey, Division of Biodiversity and Ecological Entomology, Champaign, Illinois, 61820 USA

**Keywords:** *Tsuga canadensis*, fungal epizootic, host range

## Abstract

A fungal epizootic has been detected in populations of the scale *Fiorinia externa* Ferris (Hemiptera: Diaspididae) in the eastern hemlock, *Tsuga canadensis* (L.) Carrière (Pinales: Pinaceae), of several northeastern states. *Colletotrichum acutatum* Simmonds var. *fioriniae* Marcelino and Gouli var. nov. inedit (Phyllachorales: Phyllachoraceae), a well-known plant pathogen, was the most commonly recovered fungus from these infected scales. This is the second report of a *Colletotrichum* sp. infecting scale insects. In Brazil *C. gloeosporioides* f. sp. *ortheziidae* recovered from *Orthezia praelonga* is under development as a biopesticide for citrus production. *C. acutatum* was detected growing endophytically in 28 species of plants within the epizootic areas. DNA sequences of the High Mobility Box at the MAT 1–2, mating type gene indicate that *Colletotrichum* sp. isolates recovered from scale insects and plants within epizootic areas were identical. Results from plant bioassays showed that this entomopathogenic *Colletotrichum* variety grew endophytically in all of the plants tested without causing external symptoms or signs of infection, with the exception of strawberry plants where mild symptoms of infection were observed. The implications of these findings with respect to the use of this fungus as a biological control agent are discussed.

## Introduction

The eastern hemlock, *Tsuga canadensis* (L.) Carrière (Pinales: Pinaceae), a common species in forests in the northeastern United States, is in significant decline in some areas. One causal agent associated with this decline is *Fiorinia externa* Ferris (Hemiptera: Diaspididae), the elongate hemlock scale (EHS), an exotic invasive from Japan accidentally introduced in the early 1900s (Smith and Lewis 2005). Considering the value of eastern hemlock to forest biodiversity, effective methods to manage *F. externa* are needed.

In 2002 an epizootic was reported within a population of *F. externa* in the Mianus River Gorge Preserve in Bedford, NY and attributed to unidentified fungi which produced sclerotia that often completely covered the insect (McClure 2002). Several additional epizootic sites have been identified in 36 different geographical localities in New York, Pennsylvania, Connecticut and New Jersey. The most prevalent fungus isolated from these epizootics has been morphologically and molecularly identified as *C. acutatum* var. *fioriniae* var. nov. (Marcelino et al. 2008) and has shown the ability to be easily cultured in vitro (Parker et al. 2005).

Although *C. acutatum* is more commonly known as a phytopathogen (Bailey and Jeger 1992, Prusky et al. 2000), we have identified a single previously published report of *C. gloeosporioides* f. sp. *ortheziidae* causing epizootics in the scale *Orthezia praelonga* Douglas 1891 (Hemiptera: Ortheziidae), a major pest of Citrus spp. in Brazil. Research on the biological control of this pest using *C. g. ortheziidae* has been conducted in Brazil for more than 13 years. This fungal variety is currently under commercial development (Cesnik and Ferraz 2000; R. Cesnik, personal communication 2006). Infection of *F. externa* by *C. acutatum* var. *fioriniae* var. nov. inedit. [hereafter referred to as *C. a. fioriniae*], represents the second reported case of a member of this genus infecting a scale insect. *C. a. fioriniae* may function as a natural biological control for *F. externa* in eastern hemlock forests. However, it is crucial to assess its host range and degree of infectivity before it can be considered for development as a biological control tool. This paper describes the natural occurrence of this fungal variety in plants of the hemlock forest ecosystem, as well as its phytopathogenicity to several horticultural crops.

## Material and Methods

### Plant bioassays

#### Isolates

Several entomopathogenic and phytopathogenic fungi were tested to determine their ability to infect horticultural plants and eastern hemlock seedlings (Table 1). The following isolates of *C. acutatum* were tested: five entomopathogenic isolates of *C. acutatum* var. *fioriniae* obtained from pure culture lines isolated from *F. externa* in sites where the epizootic occurs; and two phytopathogenic *C. acutatum* isolates, one from a blueberry fruit (ERL1379) and one from a tomato fruit (ERL1380). For a comparison of the phytopathogenicity of *C. acutatum* var. *fioriniae* isolates with a recognized entomopathogen, one isolate of *Lecanicillium lecanii* (Zimmerman) Gams and Zare [= *Verticillium lecanii* (Zimm.) Viégas] (EHS 132) isolated from *F. externa* was included in the bioassays. Fungal isolates have been deposited at the Univ. of Vermont, Entomology Research Laboratory (UVM ERL) Worldwide Collection of Entomopathogenic Fungi, Burlington, VT) as mature mycelia (2 wk old) in potato dextrose agar medium (1 cm^2^) in cryogenic vials (8 replicates) containing 10% glycerol, at -80°C. In the 2006 bioassays, the entomopathogenic *C. gloeosporiodes*. f. sp. *ortheziidae* from Brazil (ARSEF4360), obtained from the Agricultural Research Service Entomopathogenic Fungal Collection, Cornell University, Ithaca, NY, was also included. Isolates were grown in potato dextrose agar (PDA) (39 g/l) for 10–12 days before being harvested with sterile Pasteur pipettes to obtain suspensions of the isolates, in sterile distilled water, for subsequent calibration of conidial concentrations.

#### Plants

The virulence of the above fungal isolates was tested on the following horticultural crops: pepper (*Capsicum annuum* var. New Ace, Solanaceae); tomato (*Solanum lycopersicum* var. Patio, Solanaceae); common bush snap bean (*Phaseolus vulgaris* var. Blue Lake 274, Fabaceae); strawberry (*Fragaria* x *ananassa* var. Honeoye, Rosaceae); barley (*Hordeum vulgare*, Gramineae). These plant species were selected for testing because they are reported to be highly susceptible to *C. acutatum* (Ivey et al. 2004;Dillard and Cobb 1998; Tu 1992; Horowitz et al. 2004; Martin and Skoropad 1978). All plants were grown in a greenhouse from seed in 23-cm diameter pots containing Metro-Mix 360 potting medium (Sun Gro Horticulture, www.sungro.com/index/php). Four week old plants were used for these bioassays. In addition to the horticultural plants, 4 year old eastern hemlock seedlings (Western Maine Nurseries, www.wmnurseries.com) were also tested. Only plants with no external signs of fungal infection or disease were selected for testing.

Each plant was sprayed individually to runoff with 30 ml of a suspension of 10^6^ conidia/ml^-1^ in sterile distilled water containing 0.02% Silwet L-77 as a wetting agent (Loveland Industries, Inc., www.lovelandindustries.com). Suspensions were calibrated to the correct conidial concentration per ml sterile distilled water with an Neubauer hemocytometer (Propper Mfg. Co., www.proppermfg.com) according to the protocol of www.badgerairbrush.com) operating at 1.75 kg/cm^2^ in a sterile fume hood. Controls were treated with sterile distilled water containing 0.02% Silwet L-77. After treatment, plants were incubated in a dew chamber (3.95 m high × 1.10 m wide × 1.10 m deep built with 2.5 cm diameter PVC pipe and covered with plastic) for 24 h at 22 ± 1°C according to protocols of TeBeest (1988), then transferred to a naturally illuminated greenhouse with adjusted temperature (22 ± 0.5°C), and arranged on benches in a completely randomized block design. Each treatment was replicated four times per plant species, and the complete bioassay was run once in 2005 and 2007.

Koch's postulates were performed 1 month after treatment. Four to six leaf samples (approx. 2 cm^2^) and 8 hemlock needles were excised from each plant and surface sterilized by immersion in 75% ethanol + 0.02% Silwet L-77 for 20 s (Cowles et al. 2000), followed by 5 s in sterile distilled water, 45 s in 2.5% sodium hypochlorite (NaOCl) and finally rinsed twice in sterile distilled water for 10 s. Samples were air dried and placed on PDA supplemented with penicillin (5 ml/l) and streptomycin (12.5 ml/l). Cultures were incubated in the dark at 22°C for 10 days after which re-isolation of the test fungi was attempted.

#### Hemlock phenological trial

The potential impact of the test fungi on eastern hemlock during the growing season of 2006 was assessed using 4.5 yr old (Western Maine Nurseries, Fryeberg, ME) and 1.5 yr old (Intervale Conservation Nursery, Burlington, VT) seedlings. This test was run separate to and in addition to the plant pathogenicity trial. The plants were transplanted into Metro-Mix 360 in 11.5-, and 23-cm diameter pots, for the 1.5 and 4.5 yr old seedlings, respectively, and grown outside prior to treatment.

**Table 1.**
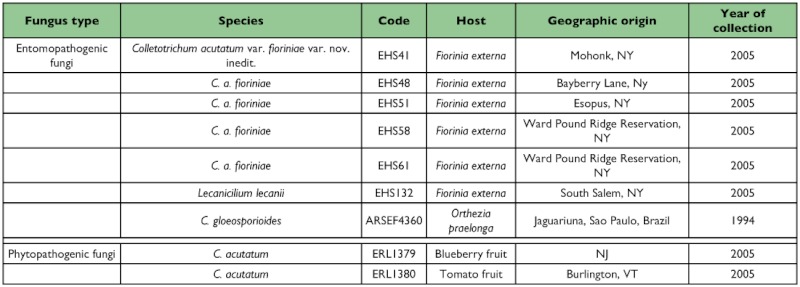
Fungal isolates used for the horticultural plant and hemlock phonological bioassays

To assess changes in potential infectivity of the fungi over the growing season, new groups of 80 seedlings (4 plants per each of the 10 fungal treatments and seedling age) were treated each month from June to September for the 1.5-yr old seedlings and May to September for the 4.5-yr old seedlings. Fungal treatments were applied as described above. After treatment, seedlings were incubated in a dew chamber for 24 h at 22 ± 0.5°C and then held in a greenhouse with ambient light and temperature ranging from 9.8 to 24°C depending on the month. Temperature was monitored using HOBO Data Loggers (Onset Computer Corporation, www.onsetcomp.com). Following treatment, seedlings were inspected monthly for symptoms or signs of disease. A single bioassay was performed during the 2006 plant growing season.

Koch's postulates were performed 1 month after treatment as described above, sampling eight needles from a randomly selected twig per seedling. This was repeated at monthly intervals throughout the test period.

#### Statistical analyses

For the horticultural plant assays, the susceptibility to fungal infection for all test plants was rated according to the probability of re-isolating all fungal isolates following treatment. The strawberry plants were used as a reference for infection because this plant is highly susceptible to infection by *C. acutatum* (Horowitz et al. 2004). The frequency of recovering a given fungal isolate was also determined for all plant types. The phytopathogenic ERL1380 was selected at random as a reference *Colletotrichum* sp. isolate to which other test isolates were compared.

All data from the bioassays were analyzed using a logistic regression analysis for binary variables. Plant species tested in the bioassays were treated as covariates. Adjusted (log10) odds ratios (with 95% confidence intervals) were calculated using SAS (SAS Institute 1990) and plotted using GraphPad software (Motulsky 1999). A Wald *χ*^2^ test was used to determine significant differences between variables (SAS Institute 1990). Treatments for which *C. acutatum* var. *firiniae* was not re-isolated were excluded from the odds ratio analysis since zeros in the denominator would result in an undefined number.

### Understory plant screening and molecular identification

To determine whether *C. acutatum* affected plants occurring in epizootic areas, samples of several common plant species were collected during 2006 from 10 localities where the *F. externa* epizootic occurred. A total of 97 plants representing 50 species growing in different strata in the hemlock forest were sampled, including low-growing shrubs, vines and trees (Table 2). A 15–20 cm branch or stem sample was taken from each plant. For the leafy plants, two to three leaves were selected at random, from which four pieces (2 cm sq.) were excised and placed in individual Petri dishes. For the hemlock needles, eight individual needles were excised and placed in separate dishes. In addition to the live material, hemlock and Tulip tree leaves (*Linodendrum tulipifera*) were also sampled from the litter. All samples were held in an incubator at (22 ± 0.5°C) prior to processing. Samples were surface sterilized as described above and placed on PDA supplemented with penicillin (5 ml/l) and streptomycin (12.5 ml/l), held in the dark at (22 ± 0.5°C) for 7 days and then inspected for the presence of *C. acutatum* vzr. *fioriniae*. This variety was distinguished from other fungi based on morphological characteristics commonly reported to be distinctive of *C. acutatum* strains (Du et al., Peres et al. 2005, Sreenivasaprasad and Talhinhas 2005), i.e.,pinkish or gray mycelium and intensely red growth medium, conidia shape and size and rapid growth.

**Table 2.**
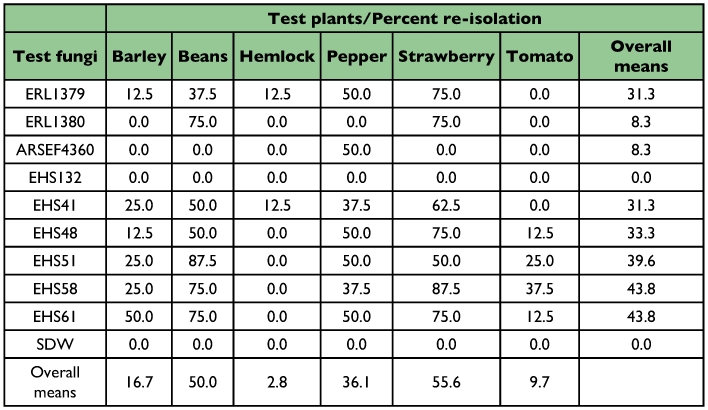
Percentage of fungal re-isolations obtained from the tested plants (N = 8 plants/isolate and plant species). No fungal re-isolations were obtained from control plants treated with SDW and Silwet

A sub-sample of 20 fungal isolates recovered from 19 different plant species were identified using molecular methods. DNA was extracted from 1-wk old cultures using the Power Soil™ DNA kit (Mo Bio Laboratories, Inc., Carlsbad, CA) following the manufacturers' directions with the following exceptions: 1) Samples were shaken for 5 min at 5.5 m/s to facilitate breakage of the cell walls using a FastPrep™ FP120 machine (Thermo Savant, www.thermo.com); 2) DNA was eluted using 100 µ″1 of diluted elution buffer (1:15) (Qiagen, www.qiagen.com), and concentrated to 20 µ″1 with a speed vacuum (Eppendorf Centrifuge 5415C, Vaudaux, www.vaudauxeppendorf.ch). The High Mobility Box (HMG) of the mating-type gene (MAT 1–2), which has been shown to differentiate between *C. acutatum* strains was amplified using primers HMGacuF and HMGacuR (Du et al. 2005).

Polymerase Chain Reaction (PCR) was conducted using Ready-To-Go PCR beads (Amersham Biosciences Inc., www.apbiotech.com) and the following protocol: initial denaturation at 95°C for 2 min followed by 30 cycles of 95°C for 30 s (denaturation), 55°C for 30 s (annealing) and 72°C for 1 min (elongation). PCR products were purified using the QIAquick® PCR purification kit (Qiagen) or Princeton Separations Centri Spin™ columns (www.prinsep.com). DNA was stored at 4°C. PCR products were sequenced using a BigDye v3 terminator cycle sequencing kit (Applied Biosystems, www.appliedbiosystems.com) with the following protocol: 95°C (initial denaturation) for 3 min followed by 30 cycles of 95°C for 10 s (denaturation), 50°C for 5 s (annealing), and 60°C for 2 min (elongation). PCR fragments were sequenced using a 3130xl Genetic Analyzer (Applied Biosystems). Chromatograms were edited and contiguous sequences were generated using Sequencher-™(Gene Codes Corp., www.genecodes.com). Primer sequences were excluded from final alignments. Sequences generated from this study were compared with MAT 1–2 gene sequences we previously obtained for the *C. acutatum* var. *fioriniae* isolates infecting *F. externa* and available from GenBank® (Accession numbers: EF593357 to EF593363). Sequences obtained in this study were also deposited in GenBank (Table 4).

**Figure 1.**
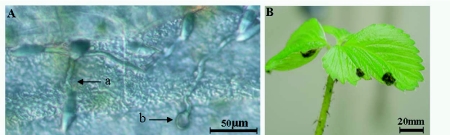
(A) Endophytic growth of *C. acutatum* var. *fioriniae* EHS48 in a bean stem 24 h after treatment, *in vitro*, showing germinated germ tubes (a) and appressoria (b); (B) strawberry with necrotic lesions, 2 mo after spraying with the *C. acutatum* var. *fioriniae* isolate.

## Results

### Plant bioassays

All treatments tested showed no signs of necrosis on plant tissue of pepper, tomato, beans, barley or hemlock. However, endophytic growth of some of the test fungi was detected by re-isolation of *C. acutatum* var. *fioriniae* isolates from asymptomatic plants after surface sterilization of leaf samples (Figure 1A, Table 2). Strawberry leaves displayed distinct local necrotrophic symptoms and endophytic growth. Although strawberry appeared to be susceptible to the *Colletotrichum* spp. treatments, the rare necrotic spots that formed (approx. 1 cm diameter) remained small and did not compromise the viability or growth of the plants 2 months post treatment, which presented the same vitality as the controls (Figure 1B).

The percentage of fungal re-isolations per plant species was not significantly different between test years, allowing data across years to be combined (Wald *χ*^2^ = 0.49, *P* = 0.48). Differences in the percentage of plants from which *Colletotrichum* spp. was re-isolated were significant among the test fungi (Wald χ2 = 14.72, *P* = 0.03), and a statistically significant 2-way interaction between the test fungi and plant species was detected (Wald *χ*^2^ = 71.02, *P* < 0.0001). Isolate EHS132 (*Lecanicilium lecanii*) and sterile distilled water were excluded from the odds ratio analysis because *Colletotrichum* spp. was not re-isolated from these treatments. Approximately 38% of the plants treated with *C. acutatum* var. *fioriniae* (average of *C. acutatum* var. *fioriniae* re-isolation) became infected (Table 2). This was comparable to the phytopathogenic *C. acutatum* from blueberry (31.3%), but significantly different from the *C. acutatum* from tomato, and the entomopathogenic *C. gloeosporioides* f. sp. *ortheziidae* isolate (*P* <0.05).

**Figure 2.**
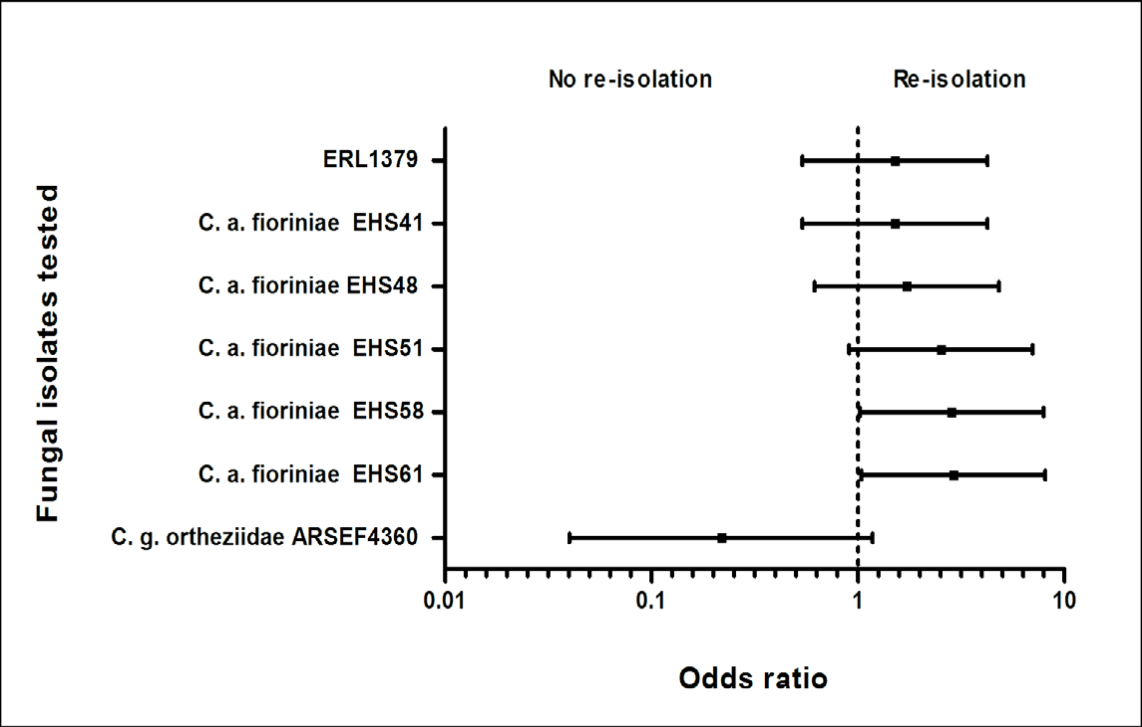
Adjusted odds ratios (95% confidence interval) of endophytic re-isolations obtained with each fungal inoculation treatment in the plant bioassays. The phytopathogenic *Colletotrichum acutatum* isolate (ERL1380) from a tomato fruit was used as the reference isolate. An odds ratio of 1 indicated no difference between the probability of re-isolating the test fungi and reference isolate; ≥1 indicated a greater probability of re-isolating the test fungi than the reference isolate; <1 indicated a greater probability of re-isolating the reference isolate than the test fungi. Confidence intervals that overlap the reference line (1) are not significantly different (*P* = 0.05).

For six of the test fungi, the mean odds ratios comparing the number of fungal re-isolations from treated plants with the reference strain, ERL1380, were > 1, indicating that they were more likely to be re-isolated from the test plants than the reference strain (Figure 2). The odds ratio for the *C. gloeosporioides ortheziidae* isolate was <1, suggesting that it was less likely to be re-isolated than the other fungi. Because of the variability in the results, differences between this isolate and four of the others were not significant. The odd ratios demonstrated that two of the *C. acutatum* var. *fioriniae* isolates were significantly different from the others isolated, indicating that these two were more infective than the reference isolate.

**Figure 3.**
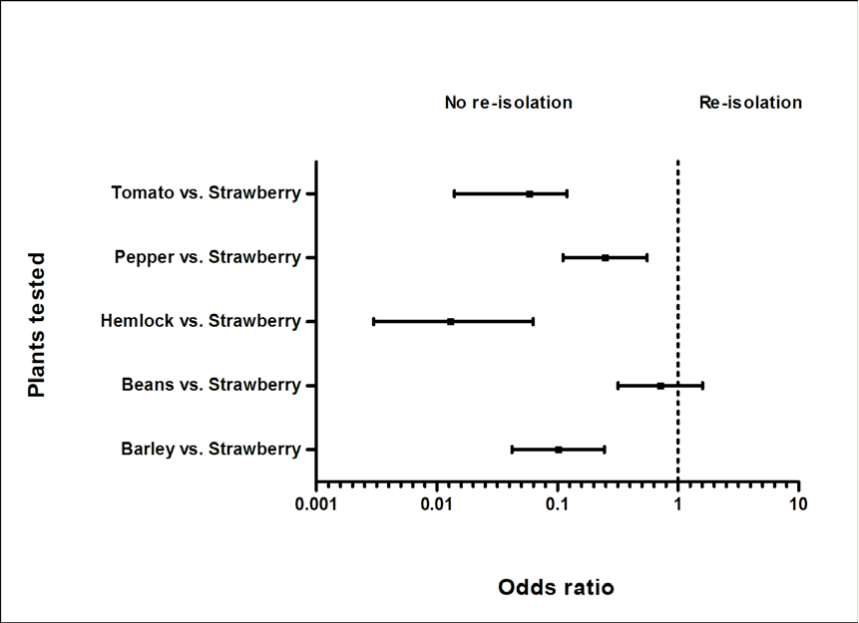
Adjusted odds ratios (95% confidence interval) of endophytic re-isolations for each plant tested in the plant bioassays. Strawberry was used as the reference plant. An odds ratio of I indicated no difference between the probability of re-isolating the fungus from the test plant species and reference plant; ≥1 indicated a greater probability of re-isolating the fungus from the test plant species than the reference species; ≤1 indicated a greater probability of re-isolating the fungus from the reference plant species than the test plants. Confidence intervals that overlap the reference line (1) are not significantly different (*P* = 0.05).

The mean odds ratios for test plants were all <1, indicating that it was less likely to recover *Colletotrichum* spp. from these plants than the reference species (strawberry) (Figure 3). Based on the percentage of plants from which *Colletotrichum* spp. was re-isolated, based on the presence of endophytic fungal growth, beans and strawberries were the most susceptible while hemlock and tomato were the least (Table 2).

### Hemlock phenological trials

**Figure 4.**
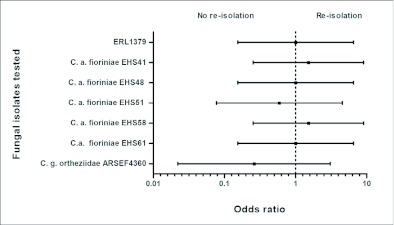
Adjusted odds ratios (95% confidence interval) of endophytic re-isolations obtained with fungal treatments in the phenological bioassays. The phytopathogenic *Colletotrichum acutatum* isolate (ERL 1380) from tomato was the reference isolate. An odds ratio of 1 indicated no difference between the probability of re-isolating the reference isolate and the test fungi; ≥1 indicated a greater probability of re-isolating the test fungi than the reference isolate; ≤1 indicated a greater probability of reisolating the reference isolate than the test fungi. Confidence intervals that overlap the reference line (1) are not significantly different (*P* = 0.05).

A significant statistical effect was found across months (Wald *χ*^2^ = 12.73, *P* = 0.01) and age of plants (*χ*^2^ = 11.53, *P* < 0.001) with respect to the response of hemlock seedlings to fungi over the growing season. The highest percentage of re-isolations occurred in July among the 1.5 year-old seedlings, with 25–100% recovery of *Colletotrichum* spp. No fungal re-isolations were recovered from 1.5 and 4.5 year-old hemlock seedlings treated in June, nor in the 4.5 year-old treated in the month of July. Isolate EHS132 was never re-isolated from hemlock seedlings and excluded from the analysis (Table 3). Based on the odds ratio analysis (Figure 4), differences in the rate of fungal recovery among the test isolates were not significant (Wald *χ*^2^ = 2.92, *P* = 0.089).

**Table 3.**
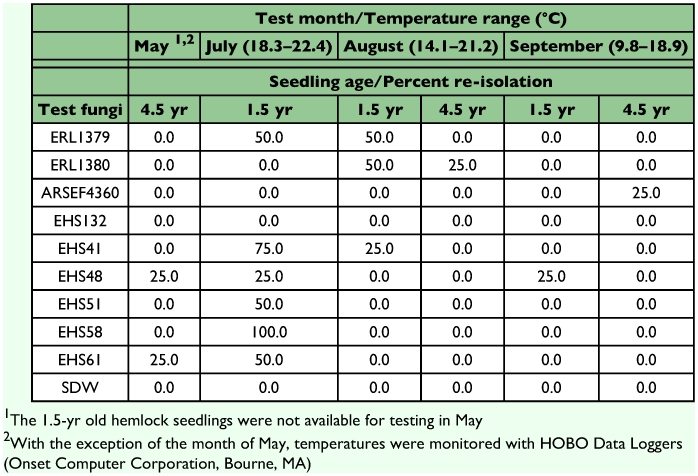
Percentage of eastern hemlock seedlings from which fungi were re-isolated after treatment (N = 4 seedlings/age and month)

Statistical differences were detected in the susceptibility of the different age of hemlock seedlings tested (Wald *χ*^2^ = 11.53, *P*<0.001). The 1.5 year-old hemlock seedlings were more susceptible to infection than the 4.5 year-old seedlings in the months of July and August. In addition. the timing of application influenced infection, with significantly greater likelihood of infection occurring when plants were sprayed in July than the other months tested. No symptoms of infection or negative impact on seedling growth were observed among the test seedlings.

### Understory plant screening and molecular identification

Of the 97 plants sampled within areas of the epizootic, 37 plants comprising 28 species in 18 families (52% of sampled species) were found to be positive for the presence of *Colletotrichum* spp. (Table 4). The High Mobility Box at the MAT1–2 gene was sequenced for twenty plant-derived isolates from eight locations where the scale epizootic occurred and found to be identical to the *C. acutatum* var. *fioriniae* strain isolated from *F. externa* (Table 4).

## Discussion

The research presented in this paper assessed the host plant associations of *Colletotrichum* spp. recovered within areas where a fungal epizootic occurs in the scale insect *F. externa*. The most commonly retrieved fungus from diseased or mummified insects in all sampled sites was molecularly and morphologically identified as *Colletotrichum acutatum* var. *fioriniae* var. nov. inedit (Marcelino et al., 2008).

With the exception of strawberry, no external signs of fungal infection were observed on the plant species tested. However, evidence of *Colletotrichum* spp. growing endophytically was commonly observed, but with some differences. Whereas *C. acutatum* var. *fioriniae* isolates were consistently recovered growing endophytically in barley, beans, pepper, strawberry and tomato, only one isolate was recovered from hemlock. In contrast, *C. gloeosporioides* f. sp. *ortheziidae* was recovered only from pepper plants.

Strawberry was found to be the most susceptible to infection by *C. acutatum* var. *fioriniae* isolates, exhibiting both external necrotrophic and endophytic growth. However, the necrotic lesions occurred sporadically among test plants, and no negative impact on plant growth was observed. In general the necrotic leaf spots did not exceed 1 cm diameter and no conidial masses were produced on the leaf surface after 2 months. *Colletotrichum* spp. commonly cause anthracnose disease in strawberries and are a serious concern for the strawberry industry (Horowitz et al. 2004). The phenotype that we observed in the strawberries exposed to *C. acutatum* var. *fioriniae* isolates is similar to the non-pathogenic strains of this genus previously reported in strawberries (Horowitz et al. 2002). Disease symptoms described for pathogenic *C. acutatum* are more varied and aggressive and include wilt, anthracnose and progressive necrosis (Sreenivasaprasad and Talhinhas 2005).

In the hemlock phenological bioassays, *C. acutatum* var. *fioriniae* isolates were commonly recovered from 1.5 yearold seedlings treated in July. The temperature during this month (diurnal average 22°C) may have been ideal for infection and colonization of these isolates. *C. acutatum* var. *fioriniae* and the phytopathogenic *C. acutatum* isolates were recovered sporadically at other times of the growing season. Thus, young seedlings of eastern hemlock may be endophytically affected by *C. acutatum* var. *fioriniae* depending on environmental conditions such as temperature. Previous accounts of both *C. acutatum* and *C. gloeosporioides* species, affecting 70 day old and 4 month old seedlings of western hemlock, *Tsuga heterophylla* (Raf.) Sarg. reported only temporary stunting resulting from *C. acutatum* infection (Lock et al. 1984, Hopkins et al. 1985), however, seedlings recovered within the first year (Canadian Food Inspection Agency 1997). In our assay temporary stunting was not observed.

*C. acutatum* var. *fioriniae* infecting the scale insect *F. externa* was also found to endophytically affect a wide variety of plants sampled in the epizootic areas. This conclusion was drawn from a comparison of DNA sequences of the High Mobility Box at the MAT 1–2 gene from *C. acutatum* var. *fioriniae* retrieved from *F. externa* and strains obtained from more than 20 plants representing a broad range of species. The High Mobility Box at the MAT 1–2 gene, which is the most variable of the genes currently in use to explore taxonomic relationships within the *C. acutatum* complex of species (Du et al. 2005), was found to be identical for both insect and plant derived *C. acutatum* isolates.

Our pathogenicity trials using plants known to be potential hosts of *Colletotrichum*, showed that *C. acutatum* var. *fioriniae* induced only mild pathogenicity in one of the six plants tested. This low degree of pathogenicity, observed under ideal fungal growth conditions, and the endophytic behavior found in the remaining test plants as well as a wide variety of plants in the epizootic area, lead us to conclude that the lack of infectivity in plants, may in part be due to the inability of *C. acutatum* var. *fioriniae* to garner sufficient nutrients to complete its life cycle using plant tissue as a host.

The wide spread presence of *C. acutatum* var. *fioriniae* in plants within the epizootic area may facilitate the exposure and subsequent infection of *F. externa* to this fungus. In this environment an opportunistic host switch may have occurred because this insect may provide a richer nutrient medium for this *C. acutatum* variety (Spatafora 2007). Moreover, the widespread occurrence of *C. acutatum* var. *fioriniae* in plants within epizootic areas may contribute to establishing a self-sustaining infection in the *F. externa* population.

Recently it has been suggested that what are considered specialized monomorphic groups of *Colletotrichum* may constitute distinct lineages of unknown recent origins that may inhabit niches other than plants (Zhu and Oudemans 2000; Guerber and Correll 2001; Peres et al. 2005; Crouch et al. 2006) and that *C. acutatum* complex of species have the ability to colonize new hosts (Sreenivasaprasad and Talhinhas 2005). Our results, which report a *C. acutatum* infecting *F. externa* and causing an epizootic, support this hypothesis. It appears that *Colletotrichum acutatum* var. *fioriniae* var. nov. inedit, can inhabit niches other than plants, in this case an insect species.

**Table 4.**
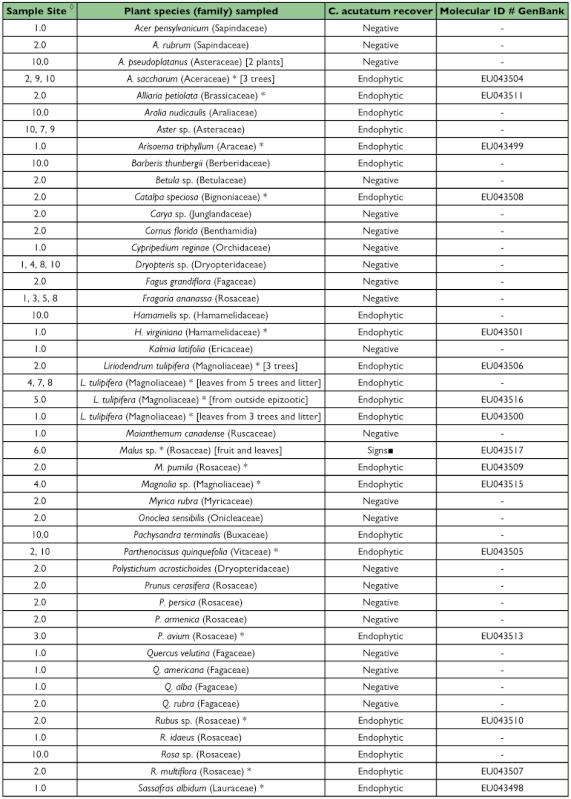
Plant material sampled in epizootic areas and screened for *C. ocutotum* (a sample of live leaves from one plant per species was taken unless otherwise indicated)

**continued:**
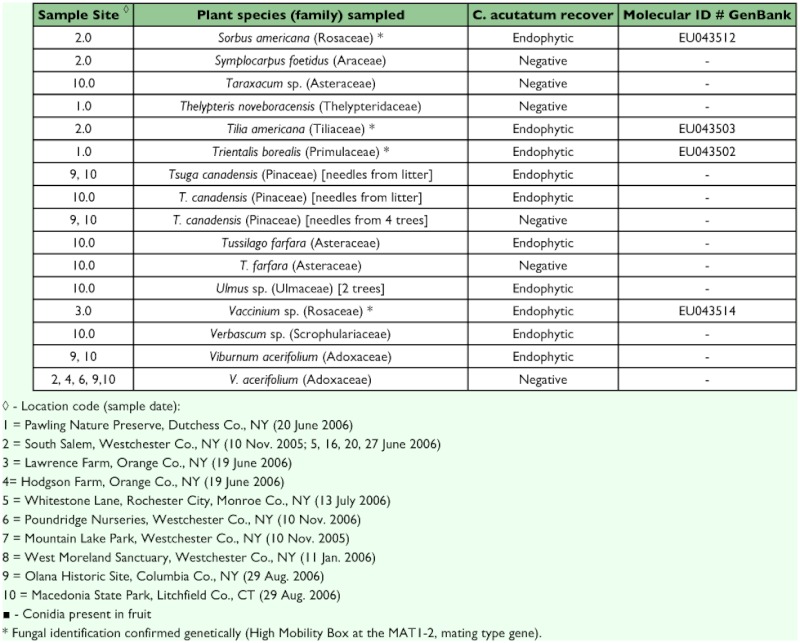

Before *C. acutatum* var. *floriniae* can be considered as a possible biological control agent for area-wide use against *F. externa*, additional research is needed to further evaluate its host range, and the etiology of the disease states triggered by this newly described *C. acutatum* variety.

## **Abbreviations**

EHS: elongate hemlock scale
